# Association between feeding practices and weight status in young children

**DOI:** 10.1186/s12887-015-0418-4

**Published:** 2015-08-26

**Authors:** Jing-Qiu Ma, Li-Li Zhou, Yan-Qi Hu, Shan-Shan Liu, Xiao-Yang Sheng

**Affiliations:** Department of Child and Adolescent Healthcare, Xinhua Hospital Affiliated to Shanghai Jiaotong University School of Medicine, MOE-Shanghai Key Laboratory of Children’s Environmental Health, Shanghai Institute for Pediatric Research, Shanghai Key Laboratory of Pediatric Gastroenterology and Nutrition, No.1665, Kongjiang Road, Yangpu District, Shanghai, 200092 China

**Keywords:** Children, Feeding practices, Overweight

## Abstract

**Background:**

Inappropriate feeding practices during infancy may lead to overweight. The aims of this study are to investigate the growth of children in the first 18 months of life; to evaluate the feeding practices of caregivers using developed Young Child Feeding Questionnaire; and to investigate caregivers’ feeding attitudes and behaviors associated with infants’ weight status.

**Methods:**

Six month-old infants and their main caregivers entering the Kongjiang Community Health Center for a routine well-child check were recruited for this study and followed up every 6 months for 12 months. Questionnaire survey was carried out through on-site face-to-face interview at each visit with the main caregivers of children using Young Child Feeding Questionnaire, which included caregivers’ feeding attitudes and behaviors. The weight and length of children were measured at each visit.

**Results:**

Among 197 children who completed the investigation at 18 months of age, 64 (32.49 %) children were overweight (BMI-for-age z scores > +1). The increases in weight-for-age z scores and BMI-for-age z scores from birth to 6 months, 12 to 18 months and birth to 18 months in overweight children were significantly higher than those in normal weight children (*P* < 0.001). In normal weight children, caregivers worried more about children’s being “underweight” and “eating less” (*P* = 0.001), whereas caregivers with overweight children worried more about children’s “eating too much” and being “overweight” (*P* < 0.001). In 64 overweight infants, the scores of “concern about child’s food intake” were significantly correlated with increase in BAZ between 12 and 18 months (*Bata* = 0.293, *P* = 0.029).

**Conclusions:**

Young Child Feeding Questionnaire is a valid tool for evaluating feeding practice of caregivers. The rapid BMI gain in overweight children may be associated with some inappropriate feeding attitudes and behaviors of caregivers.

**Electronic supplementary material:**

The online version of this article (doi:10.1186/s12887-015-0418-4) contains supplementary material, which is available to authorized users.

## Background

In recent decades, the prevalence of overweight and obesity has increased significantly in the population, especially in children, which is becoming a considerable public health burden in developed countries or in developing countries [1–3]. In Shanghai, the prevalence of obesity among children aged 0 to 6 years has risen from 2.62 % in 1996 to 6.07 % in 2002.

Overweight and obesity have adverse effects on children’s health. Moreover, childhood overweight often tracks into adulthood and is associated with the development of adverse health outcomes in late adulthood. Rapid weight gain in infancy is an important predictor of obesity in later childhood. Evidences show that rapid weight gain in early life may be closely related to the development of cardiovascular and metabolic diseases in adulthood [4–6].

Because of the lack of autonomy in early life, infants and young children are completely dependent on their caregivers. Generally, when to eat, what and how much infants and young children eat is decided by their caregivers. Therefore, the feeding patterns, feeding attitudes, feeding behaviors of main caregivers, as well as their own educational attainment, eating habits, economic status are likely to affect the growth and self-feeding patterns of infants and young children [7–9]. Apropriate feeding practices of caregivers may foster healthy eating behaviors in infants and young children and promote proper weight gain. Conversely, improper feeding practices are likely to result in inappropriate weight gain [10]. Pressure to eat or food restriction may have adverse effect on the infants’ responsiveness to internal cues of hunger and satiety, weakening their ability to self-regulate food consumption, and putting them at the risk of being overweight [10, 11]. While some studies found that responses to feeding in infants and young children and their growth status may also affect the feeding attitudes and behaviours of caregivers [12–14], other studies found no relationship between feeding practice and weight status [15].

Questionnaire investigation was a common method to evaluate the feeding practices of caregivers. Baughcum and colleagues developed the Infant Feeding Questionnaire (IFQ) related to early childhood overweight which was validated as a useful tool to assess the feeding practices of caregivers. Results suggested that there was no particular feeding style associated with overweight in young children [16]. However, in the assessment of the feeding practices in low-income African-American mothers using infant Feeding Style Questionnaire (IFSQ) which included five feeding styles, laissez-faire, restrictive, pressuring, responsive and indulgent, researchers found that pressuring or restrictive feeding style associated with energy intake and infant size was probably an important environmental factor in the development of obesity [17, 18]. These seemingly contradictory results may be due at least in part to different settings and measurements. Because feeding attitudes and behaviors may differ by cultural and socioeconomic status, feeding questionnaire as an instrument should be adapted and validated in different populations.

The aims of our study are to (1) investigate the growth of children 6–18 months; (2) evaluate the feeding practices of caregivers of children 6–18 months using developed Young Child Feeding Questionnaire (YCFQ) based on existing infant and child feeding questionnaires [16, 19, 20] combined with clinical experience; (3) investigate caregivers’ feeding attitudes and behaviors associated with infants’ weight status.

## Methods

### Study design and participants

This study was conducted in Kongjiang community, a typical community in downtown area of Shanghai. Kongjiang Community Health Center provides routine basic medical services to the local community, including well-child checks for infants and young children.

From November 2008 to April 2010, all 6-month-old infants and their parents or main caregivers receiving routine well-child check from Kongjiang Community Health Center were recruited in this study. Inclusion criteria for infants were healthy singleton infants, 6-month-old (5- to 7- month-old), born between 37 and 42 weeks gestational age, with birth weight between 2500 to 4000 g, and without metabolic or physical problems.

A total of 295 infants aged 5–7 months met the inclusion criteria. Eighteen mothers refused to participate, so 277 infants and their parents or main caregivers were included in the study and followed-up every 6 months for 1 year, at around age 12 months (11–13 months) and 18 months (17–19 months). Informations of the main caregiver’s education, mother’s pre-pregnancy weight and height, and family income were collected at baseline baseline Additional file 1. Child feeding practices including feeding environment, feeding attitudes and behaviors were also assessed in follow-up questionnaire Additional file 2 at ages of 12 and 18 months. Anthropometrics were obtained at each visit.

Of the 277 infants, 80 children had incomplete data at age 18 months, 17 caregivers refused to complete the questionnaire due to a lack of time or impatience and 63 infants had missing information in the questionnaire. Only children with complete data at 6-month baseline questionnaire and at follow-up questionnaire at 18 months, and anthropometric data of 3 visits were included in the analysis, leaving a total of 197 children. Independent sample *t* test indicated that there were no differences in child age (*P* = 0.060), body mass index (BMI, in kg/m^2^) (*P* = 0.589), BMI-for-age z scores (BAZ) (*P* = 0.538), length-for-age z scores (LAZ) (*P* = 0.325) or Weight-for-age z scores (WAZ) (*P* = 0.287) of the 80 children excluded versus the 197 children who completed the study at age 18 months.

Infants were enrolled after parents or main caregivers provided informed consent. The study protocol was approved by the Ethics Committee of Xinhua Hospital, affiliated to Shanghai Jiao Tong University School of Medicine.

### Data collection

#### Anthropometry

The weight and length of children at 6, 12 and 18 months of age were measured using standardized procedures at the community health center. Wearing light clothing, weight was measured using an electronic pediatric scale to the nearest 0.005 kg, and length was measured using a pediatric-length board to the nearest 0.1 cm with the child in a recumbent position. Standardization exercises for inter- and intra-measurer reliability in weight and length were performed in which each child was measured twice. If the differences between two measurements in weight were more than 10 g, and differences in recumbent length more than 0.4 cm, a third measurement was taken. The first or last record was reported. The birth weight and length were obtained from the infant’s birth record. Mother’s pre-pregnancy BMI was based on self-reported pre-pregnancy height and weight, and was calculated as weight in kilograms divided by the square of the height in meters (kg/m^2^).

Anthropometric indices such as WAZ, LAZ, BMI and BAZ of children at birth, 6, 12 and 18 months of age were calculated according to the 2006 WHO Child Growth Standards using the WHO Anthro 2009 software [21].

Underweight, stunting, wasting were defined as WAZ < −2, LAZ < −2, and BAZ < −2, respectively. Overweight was defined as BAZ > +1, obese was defined as BAZ > +2 [1]. Normal weight was defined as −1 ≤ BAZ ≤ +1.

After three children with BAZ < −1 were excluded, remaining 194 children aged 18 months were divided into overweight (BAZ > +1) group (*n* = 64) and normal weight (−1≦BAZ≦ + 1) group (*n* = 130). Overweight (BAZ > +1) and obese (BAZ > +2) children aged 18 months were combined into one group labeled as overweight because of the small number of obese children.

#### The Young Child Feeding Questionnaire (YCFQ)

Questionnaire items were developed from existing infant or child feeding questionnaires [16, 19, 20] and based on clinical experience of feeding attitudes and behaviors which were related to overweight in early childhood. The final questionnaire included 38 items Additional file 3.

The items of feeding practices were scored on a 5-point Likert-type scale. The rating scales were anchored by the terms “never” and “always” (responses were recorded using the following scale: 1 = Never, 2 = Rarely, 3 = Sometimes, 4 = Often, 5 = Always).

The information of child feeding practices was obtained using the young child feeding questionnaire (YCFQ) through on-site face-to-face interviews with the parents or main caregivers by a trained clinical research assistant at each visit.

### Statistical analysis

SPSS 13.0 statistical software was used for all descriptive analyses. We conducted exploratory factor analysis (varimax rotation) on the 38 items to determine the underlying factor structure of the YCFQ. Factors with eigenvalues greater than 1.0 were retained, and individual item was retained if it loaded above 0.30 on a single factor. Factor analysis was repeated three times to determine all items that met these loading criteria. Internal consistency of each factor was assessed by Cronbach’s alpha (*α*). Pearson correlation coefficient (*r*) was used if a factor had only two items. The scores of the items in each factor were summed to obtain a total score of each factor. The score medians (interquartile range) of each item and each factor were calculated. The inter-factor correlations were tested by Spearman correlation.

Measurement data were presented as mean ± SD for normally distributed continuous variables or median (interquartile range) for data not normally distributed. Categorical data were summarized by calculating percentages. Anthropometric z scores such as WAZ, LAZ and BAZ at birth, 6, 12 and 18 months of age between normal weight and overweight children were compared by analysis of covariance after adjusting for birth weight and mothers’ pre-pregnancy BMI. Factor scores between normal weight and overweight children were compared by Wilcoxon rank sum test. The chi-square test was used to examine the differences in characteristics of the main caregivers between normal weight and overweight children. The linear mixed model was used to analyse the growth trends of the children from birth to 18 months and the differences were compared between overweight and normal weight children. The associations between factor scores of YCFQ and the increase in BAZ from 12 to 18 months in overweight children were examined by a multiple linear regression analysis after adjusting for birth weight and mothers’ pre-pregnancy height as possible confounding variables.

All statistical tests were two-tailed and *P* values <0.05 were considered statistically significant.

## Results

### Discription of study sample

The mean age of 197 children who completed the 12-month follow-up was (18.11 ± 0.27) months. Of those, 93 were boys (47.16 %) and 104 were girls (52.79 %). The anthropometric indices of 197 children from birth to 18 months are shown in Table 1. Most children grew well in this community. Evaluated by the WHO Child Growth Standards, mean LAZ, WAZ and BAZ of 197 children at 18 months were all above the WHO median. There was only one child (0.51 %) who exhibited sign of wasting (BAZ < −2) and there was no underweight (WAZ < −2) or stunting (LAZ < −2). The BAZ of three children (1.52 %) were less than −1. However, 64 children (32.49 %), including 33 boys and 31 girls were overweight (BAZ > +1). Among them, 9 (4.57 %) children, including 3 boys and 6 girls were obese (BAZ > +2). There was no significant difference in the prevalence of overweight between girls and boys (35.48 % vs. 29.81 %, *χ*^*2*^ = 0.721, *P* = 0.396). The difference in prevalence of obesity by gender was not significant (5.77 % vs. 3.23 %, *χ*^*2*^ = 0.262, *P* = 0.609).

**Table 1:** Growth from birth to 18 months in normal weight and overweight children

Items	Normal weight children (*n* = 130)	Overweight children (*n* = 64)	Overall (*n* = 197)	Statistical value (*F*)	*P* ^*a*^
Weight-for-age z scores (WAZ)					
At birth	0.02 ± 0.66	0.12 ± 0.66	0.06 ± 0.66	0.700	0.404
At 6 months	0.62 ± 0.74	1.36 ± 0.76	0.86 ± 0.82	38.979	<0.001
At 12 months	0.49 ± 0.69	1.28 ± 0.65	0.75 ± 0.77	55.388	<0.001
At 18 months	0.31 ± 0.63	1.44 ± 0.51	0.66 ± 0.81	148.952	<0.001
Increase in WAZ					
0-6 months	0.60 ± 0.80	1.24 ± 0.85	0.81 ± 0.88	39.400	<0.001
6-12 months	−0.13 ± 0.47	−0.07 ± 0.59	−0.11 ± 0.51	0.589	0.444
12-18 months	−0.18 ± 0.64	0.16 ± 0.49	−0.09 ± 0.63	15.303	<0.001
0-18 months	0.29 ± 0.76	1.32 ± 0.76	0.60 ± 0.93	160.586	<0.001
Length-for-age z scores (LAZ)					
At birth	0.03 ± 0.64	−0.04 ± 0.62	0.00 ± 0.63	0.945	0.332
At 6 months	0.48 ± 0.80	0.72 ± 0.93	0.57 ± 0.85	3.447	0.065
At 12 months	0.41 ± 0.89	0.56 ± 0.91	0.46 ± 0.90	1.529	0.218
At 18 months	0.32 ± 0.93	0.56 ± 0.86	0.41 ± 0.92	3.258	0.073
Increase in LAZ					
0-6 months	0.45 ± 0.94	0.76 ± 0.96	0.56 ± 0.95	4.770	0.030
6-12 months	−0.08 ± 0.62	−0.16 ± 0.70	−0.10 ± 0.64	0.406	0.525
12-18 months	−0.09 ± 0.76	−0.00 ± 0.68	−0.06 ± 0.73	0.579	0.448
0-18 months	0.28 ± 1.09	0.60 ± 0.90	0.40 ± 1.04	4.561	0.034
BMI-for-age z scores (BAZ)					
At birth	0.02 ± 0.97	0.23 ± 0.88	0.09 ± 0.94	2.041	0.155
At 6 months	0.47 ± 0.77	1.27 ± 0.93	0.72 ± 0.91	36.907	<0.001
At 12 months	0.40 ± 0.64	1.28 ± 0.73	0.68 ± 0.80	69.581	<0.001
At 18 months	0.19 ± 0.49	1.56 ± 0.45	0.61 ± 0.85	335.717	<0.001
Increase in BAZ					
0-6 months	0.45 ± 1.18	1.03 ± 1.16	0.63 ± 1.22	23.086	<0.001
6-12 months	−0.07 ± 0.62	0.02 ± 0.69	−0.04 ± 0.64	0.738	0.391
12-18 months	−0.20 ± 0.71	0.28 ± 0.64	−0.07 ± 0.76	22.459	<0.001
0-18 months	0.18 ± 1.05	1.33 ± 1.01	0.51 ± 1.22	174.607	<0.001

^*a*^Analysis of covariance by adjustment of mother’s pre-pregnancy BMI for WAZ, LAZ and BAZ at birth, and by adjustment of birth weight and mother’s pre-pregnancy BMI for other variables

### Comparison of growth from birth to 18 months between overweight and normal weight children

There was no significant difference in WAZ, LAZ and BAZ at birth between normal weight (−1≦BAZ≦ + 1, *n* = 130) and overweight (BAZ > +1, *n* = 64) children at 18 months after adjusting for mothers’ pre-pregnancy BMI (*P* = 0.404, 0.332 and 0.155). The WAZ and BAZ at age 6, 12 and 18 months in overweight children were all significantly higher compared with normal weight children after controlling for birth weight and mothers’ pre-pregnancy BMI (*P* < 0.001). However, no significant difference in LAZ at age 6, 12 and 18 months existed between normal weight and overweight children (*P* = 0.065, 0.218 and 0.073) (Table 1).

The increases in WAZ and BAZ from birth to 6 months, 12 to 18 months and birth to 18 months in overweight children were significantly higher than those in normal weight children after adjusting for birth weight and mothers’ pre-pregnancy BMI; this difference was not present for 6–12 months (*P* < 0.001) (Table 1).

Linear mixed model was used to analyse the growth tendencies of 194 children (3 children with BAZ < −1 were excluded) from birth to 18 months and found that WAZ, LAZ and BAZ of all children changed significantly with age (age 0, 6, 12 and 18 months) (*F* = 56.586, *P* < 0.01; *F* = 15.338, *P* < 0.01; *F* = 40.891, *P* < 0.01). Furthermore, the variation tendencies of WAZ, LAZ and BAZ from birth to 18 months in overweight children were significantly different from those in normal weight children (*F* = 150.925, *P* < 0.01; *F* = 4.697, *P* = 0.031; *F* = 181.742, *P* < 0.01).

### Main caregivers

As far as the main caregivers were concerned, among 197 children, 72 (36.55 %) children were mainly fed by grandparents, 62 (31.47 %) were mainly fed by parents, 61 (30.96 %) were fed jointly by grandparents and parents, and 2 (1.02 %) were fed by relatives at the 18-month visit. Years of education less than 9 was found in the maincaregivers of 36 children (18.27 %), 9 ~ 12 years in maincaregivers of 42 children (21.32 %) and more than 12 years in maincaregivers of 119 children (60.41 %). There were 65 children (32.99 %) whose annual household income was less than RMB 60,000 per year, 85 (43.15 %) with annual household income of RMB 60 ~ 150,000 per year, and 47 (23.86 %) with annual household income of RMB more than 150,000 per year.

Excluding three 18-month-old children with BAZ < −1, the maternal pre-pregnancy BMI of overweight children (*n* = 64) was significantly higher than that of normal weight children (*n* = 130) (21.92 ± 2.93 vs. 20.85 ± 2.81, *t* = −2.470, *P* = 0.014), however, there were no significant differences between normal weight children and overweight children in the composition of main caregivers (*Fisher’s exact test*, *P* = 0.626), in years of education of main caregivers (*χ*^*2*^ = 3.774, *P* = 0.152), and in annual household income (*χ*^*2*^ = 2.086, *P* = 0.352) (Table 2).

**Table 2:** Characteristics of the main caregivers

Items	Normal weight children	Overweight children	Statistical value (*χ* ^*2*^)	*P*
	(*n* = 130)	(*n* = 64)		
Composition [*n* (%)]				
Parents	38 (29.23)	24 (37.50)	—	0.626^*a*^
Grandparents	49 (37.69)	22 (34.38)		
Parents and grandparents	41 (31.54)	18 (28.13)		
Others	2 (1.54)	0 (0.00)		
Years of education [*n* (%)]				
≦9 years	29 (22.31)	7 (10.94)	3.774	0.152
9-12 years	26 (20.00)	16 (25.00)		
> 12 years	75 (57.69)	41 (21.65)		
Annual household income [*n* (%)]				
≦RMB 60 thousand per year	39 (30.00)	25 (39.06)	2.086	0.352
RMB 60–150 thousand per year	57 (43.85)	27 (42.19)		
> RMB 150 thousand per year	34 (26.15)	12 (18.75)		

Three young children with BAZ < −1 at 18 months were excluded

^*a*^Fisher’s exact test

### Factor structure

Factor analysis resulted in a ten-factor solution for the final 28 items retained (Table 3). The accumulated variance contribution rate of the final factor structure was 68.56 % and communalities for these 28 items ranged from 0.45 to 0.86. Three factors (Factor 1, 2 and 4) included 4 items, two (Factor 3 and 5) included 3 items, and five (Factor 6, 7, 8, 9 and 10) included 2 items. According to the contents of the items composing each factor, these ten factors were named. The contents of items composing Factor 1 and 2 reflected the feeding attitudes of main caregivers, and Factor 1 and 2 were named “concern about child undereating or being underweight” and “concern about child overeating or being overweight”, respectively. Factor 4, 8, 9 and 10 focused on the feeding behaviors of main caregivers, and were named “over-feeding behavior”, “pushing the child to eat more”, “using food to calm the child” and “concern about child’s food intake”, respectively. Factor 3 and 6 named “interaction with the child during feeding” and “language communication while feeding” reflected the feeding environment created by caregivers. Factor 5 was named “children’s food preferences” concerning about the degree of preference in children for calorie-dense foods, puffed foods, fried foods, starchy foods, carbonated beverages, and so on. Factor 7 was named “awareness of child’s hunger and satiety cues”. Four factors (Factor 1, 2, 3 and 7) had coefficient alphas above 0.70, but the four factors with two items (Factors 6, 8, 9 and 10) each had Pearson correlation coefficients below 0.50. The ten factors were generally not highly related to each other, with inter-factor correlations ranging from 0.004 to 0.419.

**Table 3:** Factor and item descriptive statistics, factor loadings, and internal consistency for the ten-factor solution of the young child feeding practice questionnaire (*n* = 197)

Factors and items composing each factor (median, interquartile range)	Factor loading											
	α^*a*^	1	2	3	4	5	6	7	8	9	10	h^2*b*^
*Factor 1* *-Concern about child undereating or being underweight (8, 5–13)*	0.77											
1. Worried child being underweight (1, 1–4)		**0.86** ^*C*^	−0.10	−0.14	—^*d*^	0.12	—	—	—	—	—	0.79
2. Felt that one was not doing a good job in feeding when one saw that other children of the same age weighed more (1,1-2)		**0.83**	—	—	—	—	—	—	0.16	—	—	0.73
3. Upset if child did not eat enough (2, 1–4)		**0.60**	—	—	0.45	—	—	—	—	0.12	—	0.59
4. Worried child was not eating enough (3, 1–4)		**0.55**	—	—	0.40	—	—	—	0.23	—	−0.24	0.58
*Factor 2* *-Concern about child overeating or being overweight (4, 4–8)*	0.77											
5. Upset if child ate too much (1, 1–3)		—	**0.81**	—	—	−0.10	—	—	−0.22	—	—	0.75
6. Worried child was eating too much (1, 1–2)		—	**0.80**	—	—	—	−0.17	—	−0.27	—	0.15	0.77
7. Restriction of food intake to prevent the child becoming overweight		−0.21	**0.73**	—	−0.15	—	—	—	0.33	—	—	0.73
8. Worried child being overweight (1, 1–1)		−0.27	**0.70**	−0.19	—	—	—	—	0.21	—	—	0.65
*Factor 3* *-Interaction with the child during feeding (15, 13–15)*	0.76											
9. Smiled to your child while feeding (5, 4–5)		—	—	**0.85**	—	—	0.31	—	—	—	—	0.84
10. Eye contact while feeding (5, 4–5)		—	—	**0.84**	—	—	0.30	—	—	—	—	0.81
11. Face-to-face feeding (5, 5–5)		—	—	**0.72**	—	—	−0.24	—	0.14	—	—	0.63
*Factor 4* *-Over-feeding behavior (13, 9–15)*	0.54											
12. If child did not eat enough, changed feeding method to feed more (4, 1–5)		—	—	—	**0.80**	0.20	−0.15	−0.11	—	—	—	0.73
13. Diverted the child’s attention to feed more (4, 2–5)		—	—	−0.17	**0.70**	—	0.20	—	0.13	0.20	—	0.65
14. Fed more before sleep to let the child sleep longer (1, 1–5)		0.11	—	0.14	**0.44**	−0.18	−0.27	−0.21	—	0.22	−0.21	0.45
15. If child ate less at one meal, prepared other foods for him/her within 30 min (3, 1–4)		0.19	−0.26	0.13	**0.34**	0.32	0.33	—	0.11	0.10	—	0.47
*Factor 5* *- Children’s food preferences (6, 3–8)*	0.60											
16. Did your child prefer calorie-dense food (e.g. sweets, chocolate, etc.) (1, 1–3)		—	—	—	—	**0.83**	—	—	—	—	—	0.70
17. Did you give your child puffed food, candy, carbonated beverages, fried foods (1, 1–2)		—	—	0.12	—	**0.80**	—	—	—	—	−0.27	0.75
18. Did your child prefer starchy foods (cookies, cakes, bread and potatoes) (2, 1–4)		0.17	—	—	−0.11	**0.55**	0.24	0.11	−0.19	0.17	0.35	0.61
*Factor 6* *-Language communication while feeding (9, 7–10)*	0.48											
19. Talked or sang to child while feeding (4, 4–5)		—	—	—	—	—	**0.76**	0.12	0.11	—	—	0.62
20. Praising for the child’s good eating behaviors (5, 4–5)		—	—	0.22	−0.11	—	**0.72**	0.11	—	−0.17	—	0.64
*Factor 7* *-Awareness of child’s hunger and satiety cues (10, 8–10)*	0.72											
21. I knew when child was full (5, 4–5)		—	—	—	—	—	—	**0.90**	—	—	—	0.83
22. I knew when child was hungry (5, 4–5)		—	—	0.14	—	—	0.15	**0.89**	—	—	—	0.86
*Factor 8* *-Pushing the child to eat more (3, 2–5)*	0.37											
23. Regardless the child’s refusal and made him/her eat a certain amount (1, 1–3)		0.25	—	—	0.15	—	—	−0.15	**0.75**	—	—	0.67
24. Encouraging the child to eat more (1, 1–3)		0.47	—	—	0.14	—	0.22	—	**0.50**	—	—	0.55
*Factor 9* *-Using food to calm the child (2, 2–5)*	0.44											
25. Feeding child was the best way to stop fussiness (1, 1–1)		0.14	—	−0.11	—	—	−0.18	—	0.13	**0.82**	−0.10	0.78
26. When fussy, feeding child was first thing you would do (1, 1–3)		—	—	—	0.14	0.19	—	—	—	**0.78**	—	0.69
***Factor 10*** *-Concerned about child’s food intake (7, 5–8)*	0.17											
27. Could the child eat all foods you provided at every meal (3, 3–4)		−0.15	—	—	−0.15	—	—	—	—	—	**0.76**	0.65
28. Did you think your child should eat all foods you provided at every meal (4, 1–5)		0.17	—	—	0.17	—	—	—	0.44	—	**0.63**	0.66

^*a*^Measure of internal consistency: Cronbach alpha (*α*). Pearson correlation coefficient (*r*) used if only two items per factor

^*b*^h^2^ = communality

^*c*^Boldface indicates highest factor loadings

^*d*^— indicates factor loading (absolute value) was less than 0.1

### Difference in factor scores by weight status

Scores of ten factors were compared between caregivers of overweight and normal weight children and significant differences in feeding attitudes were found (Table 4). While caregivers of normal weight children were significantly more concerned about their children’ undereating or being underweight (*Z* = −3.260, *P* = 0.001) (Factor 1), caregivers of overweight children showed higher levels of concern about their children’ overeating or being overweight (*Z* = −4.035, *P* < 0.001) (Factor 2). There were no significant differences in scores of remaining 8 factors.

**Table 4:** Differences in factor scores by weight status

Factor (range)	Factor score			Statistical value (*Z*)	*P* ^*b*^
	Overall (*n* = 197)	Normal weight (*n* = 130)	Overweight (*n* = 64)		
Factor 1-Concern about child undereating or being underweight (4–20)	8(5–13)^*a*^	9.5(6–13)	7(4–11)	−3.260	0.001
Factor 2-Concern about child overeating or being overweight (4–20)	4(4–8)	4(4–8)	7(4–12)	−4.035	<0.001
Factor 3-Interaction with the child during feeding (3–15)	15(13–15)	15(13–15)	15(13–15)	−0.020	0.984
Factor 4-Over-feeding behavior (4–20)	13(9–15)	13(9–16)	12(8.25-14.75)	−1.160	0.246
Factor 5- Children’s food preferences (3–15)	6(3–8)	6(3–8)	6(3.25-8)	−0.436	0.663
Factor 6-Language communication while feeding (2–10)	9(7–10)	9(8–10)	9(7–10)	−1.159	0.246
Factor 7-Awareness of child’s hunger and satiety cues (2–10)	10(8–10)	10(8–10)	10(8–10)	−0.059	0.953
Factor 8-Pushing the child to eat more (2–10)	3(2–5)	3(2–5)	3(2–5.75)	−0.707	0.480
Factor 9-Using food to calm the child (2–10)	2(2–5)	2(2–4.25)	3(2–5)	−1.664	0.096
Factor 10-Concern about child’s food intake (2–10)	7(5–8)	7(5–8)	6(5–8)	−0.067	0.946

^*a*^median (interquartile range)

^*b*^Wilcoxon rank sum test

### Correlation of increase in BAZ between 12 and 18 months and factor scores of YCFQ in overweight children

In 64 overweight children at 18 months, the scores of “concern about child’s food intake” (Factor 10) were positively correlated with the increase in BAZ between 12 and 18 months (*Bata* = 0.293, *P* = 0.029) after adjusting for birth weight and mother’s pre-pregnancy height by multiple linear regression analysis (Table 5).

**Table 5:** Correlation between increase in BAZ between 12 and 18 months and factor scores of YCFQ in overweight children (*n* = 64)

Factor	Increase in BAZ between 12 and 18 months		
	*Beta* ^*a*^	*Std.Error*	*P* ^*b*^
Factor 1-Concern about child undereating or being underweight	0.197	0.020	0.133
Factor 2-Concern about child overeating or being overweight	−0.041	0.018	0.748
Factor 3-Interaction with the child during feeding	0.185	0.034	0.157
Factor 4-Over-feeding behavior	0.086	0.020	0.503
Factor 5- Children’s food preferences	−0.089	0.033	0.510
Factor 6-Language communication while feeding	0.061	0.043	0.643
Factor 7-Awareness of child’s hunger and satiety cues	−0.017	0.049	0.894
Factor 8-Pushing the child to eat more	0.185	0.037	0.160
Factor 9-Using food to calm the child	−0.234	0.043	0.068
Factor 10-Concerned about child’s food intake	0.293	0.040	0.029

^*a*^Standardized partial regression coefficient

^*b*^Association between increase in BAZ from 12 to 18 months and factor scores of YCFQ in overweight children adjusted for birth weight and mother’s pre-pregnancy height

## Discussion

The prevalence and severity of childhood overweight and obesity are steadily increasing and has become a global public health problem. The same situation happens in China, especially in Shanghai, an affluent metropolis [22, 23]. Rapid weight gain in infancy is probably the initiation of childhood obesity, and also contributes to the increase in risk of metabolic disorders in adulthood. However, the feeding for infants and young children almost completely depends on their caregivers, especially in infancy. The caregiver’s feeding beliefs and behaviors may have a great impact on the growth and development of infants and young children.

In our study, the prevalence of overweight in young children aged 18 months was high (32.49 %) in this community. Furthermore, the increases in WAZ and BAZ from birth to 6 months, 12 to 18 months and birth to 18 months in overweight children were significantly higher than those in normal weight children after controlling for birth weight and mothers’ pre-pregnancy BMI. A growing number of studies have confirmed that rapid early weight gain is a significant risk factor for obesity in later life [24, 25]. Weight gain during the first 24 months of life was also considered as the best overall predictor of overweight at school entry [26]. Our study indicates that approximately one-third of the infants in this community had rapid weight gain and are at the risk of becoming overweight or obesity in their later life.

A systematic review and meta-analysis revealed that pre-pregnancy overweight/obesity increased the risk of high birth weight and subsequent offspring overweight/obesity [27]. A multi-ethnic cohort study also found that maternal pre-pregnancy BMI was positively correlated to infant’s weight and BMI at 14 months [28]. Similarly, our study showed that the maternal pre-pregnancy BMI of overweight children was significantly higher than that of normal weight children. Genetic mechanism, familial characteristics such as food preferences, eating habits and lifestyle may contribute to the association between maternal pre-pregnancy overweight and offspring overweight.

Factor analysis of the YCFQ yielded ten factors consisting of 28 items, and feeding attitudes, feeding behaviors, feeding environment, food preference of children were involved in ten factors. The cumulative variance contribution rate of ten factors were 68.56 %, and the communalities for all of the items were in the range of 0.47 ~ 0.86. Therefore, it indicates that the CFQ can be a valid tool for evaluating feeding practice in our setting.

As expected, the findings of YCFQ found that caregivers of normal weight children were more concerned about the children’s undereating or being underweight, while caregivers of overweight children were more concerned about the children’s eating too much and being overweight. A similar result was reported in a study in which the parents of boys with normal BMI were more concerned about their boys’ underweight compared to the parents of boys with high BMI and thus adopted more over-feeding behaviors [29]. This suggests that the weight status of children influences the feeding attitudes of caregivers. However, there was no difference in over-feeding behaviors between caregivers of normal weight and overweight children.

Through multiple linear regression analysis, we found that the increases in BAZ from 12 to 18 months in 64 overweight children were significantly positively correlated with the scores of factor 10. It is suggested that such feeding attitude and behavior as pushing children to eat a certain amount of food is likely driving the rapid increase in BAZ from 12 to 18 months in overweight children.

In this study, grandparents played a leading or secondary role in feeding of 67.5 % children. Furthermore, the composition, education duration and annual household income of main caregivers were similar between normal weight and overweight young children. In China, grandparents often have the main responsibility for infant and young child feeding, which is different from western countries in which parents are usually the main caregivers responsible for children’s food intake. Due to rapid economic development and increased income in recent decades, nutritional status of infants and young children in China has improved greatly. In metropolitan cities and particularly in Shanghai, main nutritional problems have changed from undernutrition to overnutrition. However, in Chinese culture, there is still a widespread belief that fat represents health, especially for grandparents. Driven by this belief, caregivers, mainly composed of grandparents, commonly push children to eat a certain amount of food at every meal, regardless of children’ internal cues of hunger and satiety. Moreover, the amount of food to feed determined by caregivers according to their own standard is likely to exceed their children’s needs. This sort of feeding attitude and behavior may undermine the ability to regulate food intake in infants and children, leading to desensitization of hunger and satiety and subsequent excessive weight gain and obesity [30].

There are some notable findings in this study. The 12-month follow-up from 6 to 18 months of age showed that overweight children always had a more rapid weight gain in comparison with normal weight children at each visit. Furthermore, combining with a survey at 18 months, we found that caregivers of overweight children had some improper feeding attitudes and behaviors based on lack of awareness of internal cues of hunger and satiety which were closely related to the rapid weight gain in the last six months.

Our study has several limitations. First, the data were only collected from a community in Shanghai which may not be representative of child feeding practice in China. Secondly, because the caregivers reported about themselves with no objective reporters, the risk of reporting bias may exist.

## Conclusion

In conclusion, improper feeding attitudes and behaviors of caregivers due to lack of awareness of internal cues of hunger and satiety may result in rapid weight gain of infants and young children and increase the risk of overweight/obesity in the later life. Once obesity is established, therapeutic interventions are expensive and tend to have poor long-term effects. Infancy may be the most effective period to prevent overweight and obesity [31, 32]. Community health centers, playing an important role in the prevention of overweight and obesity, should pay more attention to health education about feeding. It is necessary to raise caregivers’ recognition of childhood overweight and correct inappropriate feeding attitudes and related risk feeding behaviors in order to prevent overweight. Future public health programs should include prospective studies on infant and young child feeding practices in an effort to identify effective preventive strategies to prevent early obesity.

## Additional files

Additional file 1:
**The baseline questionnaire.** (PDF 134 kb) Additional file 2:
**The follow-up questionnaire.** (PDF 107 kb) Additional file 3:
**The young child feeding questionnaire (YCFQ).** (PDF 122 kb)

## Abbreviations

WAZ: Weight-for-age z scores
LAZ: Length-for-age z scores
WLZ: Weight-for-length z scores
BMI: Body mass index
BAZ: BMI-for-age z scores
YCFQ: The Young Child Feeding Questionnaire
