# Supplementary material for: Association between feeding practices and weight status in young children
Source: BMC Pediatr. 2015 Aug 26;15:97. doi: 10.1186/s12887-015-0418-4 (PMC4550067; doi:10.1186/s12887-015-0418-4)
Supplement: Additional file 1: — The baseline questionnaire. (PDF 134 kb) [file 12887_2015_418_MOESM1_ESM.pdf]

上海市 5-7 月龄婴儿辅助食品添加调查 地区[________________________]

代码: |___|___|___|___|___|___|

电话：

地址 [________________________]

第一部分：一般情况

1. 孩子的出生日期： |__|__|__|__|-|__|__|-|__|__| (阳历) ☐ 男孩 ☐ 女孩
2. 填表日期： |__|__|__|__|-|__|__|-|__|__|
3. 母亲怀孕的孕周：|___|___|周+ |___|日；母亲的身高|__|___|___|. |___| cm；母亲的体重|___|___|.|___|___| kg
4. 出生时的身长： |___|___|. |___| cm
5. 出生时的体重： |___|___|___|___| g
6. 出生时有无抢救： 1 ☐ 无 2 ☐ 有 →（请说明： ）
7. 自出生至今是否患过重大疾病(是否住院治疗）：1 ☐否

   2 ☐是 （请列出： ）
8. 最近一次血常规中血红蛋白含量：|__|__|__|g/l。
9. 孩子的主要带养人是：1 ☐ 父母 2 ☐ 祖父母或外祖父母 3 ☐ 保姆 4 ☐ 其他人：
10. 主要带养人学历：1☐ 小学 2 ☐ 中学 3 ☐ 高中 4 ☐ 大专 5 ☐ 大学 6 ☐ 研究生及以上
11. 主要带养人职业：1 ☐ 立法者/公务员/经理 2 ☐ 专业人员 3 ☐ 技师/助理专业人员 4 ☐ 店员

    5 ☐ 服务/商店/市场销售 6 ☐ 车间/机器的操作管理 7 ☐ 手工艺和相关劳动者

    8 ☐ 农民或渔民 9 ☐ 军人 10 ☐ 家庭主妇 11 ☐ 不适用（原因： ）
12. 母亲的产假有多久：1 ☐ 无 2 ☐ 1 月 ~3 月 3 ☐ 4~6 月 4 ☐ 7~12 月 5 ☐ ＞12 月
13. 家庭年收入：1 ☐ 5 千元以下 2 ☐ 5 千元至 3 万元 3 ☐ 3 万元至 6 万元 4 ☐ 6 万元至 15 万元

    5 ☐ 15 万元至 30 万元 6 ☐ 30 万元至 50 万元 7 ☐ 50 万元以上

第二部分：喂养情况

1. 您的孩子除了喂母乳外，没有喂过任何其他食物或饮料有多长时间？（可以喝水）

   1☐ 从来没有喂过母乳 2☐ 0-3 月 3☐ 4-5 月

   4☐ 6-7 月 5☐ 8-9 月 6☐ 现在还只喂母乳

2. 您是否给孩子喂水？ 1☐ 是 2☐ 没有 → 跳到问题 5

3.您给孩子喂白开水还是添加果汁、蜂蜜的水？1☐ 白开水 2 ☐ 添加果汁 3 ☐ 添加蜂蜜 4 ☐ 其他

4. 您从孩子多大开始喂水？1☐ 0-1 月 2☐ 1~月 3☐ 2~ 月 4☐ 3~ 月 5☐ 4~ 月 6 ☐ ≥6 月

5. 您现在是否仍然在给孩子喂奶? 1☐ 是 2☐ 没有 → 跳到问题 7

6.您每天大约给孩子喂几次奶? |___|___|

7. 您的孩子现在是否吃或喝任何除母乳外的食物（除水外）？

   1☐ 是 2 ☐ 没有 → 跳到第三部分

8. 您每天给孩子吃或喝其他的食物（除喂母乳、钙粉、鱼肝油外）有几次？

   1☐ 1 次 2 ☐ 2 次 3☐ 3 次 4☐ 4 次或以上

上海市 5-7 月龄婴儿辅助食品添加调查　地区[________________________]

代码: |___|___|___|___|___|___|

电话：　　　　　　　　　　　　　　　　　地址 [________________________]

9. 您是否给孩子吃过您从商店买来的婴儿食品（如，米粉、婴儿奶粉）? 1□ 是　　2□ 没有 → 跳到问题 12

10.您孩子吃的是哪种购买的婴儿食品?

1□ 婴儿米粉　2□ 婴儿奶粉　3□ 婴儿米粉和婴儿奶粉　4 □　其他 (请列出) ______________

11. 如果吃这些购买的婴儿食品，多久吃一次? 1□ 几乎每天　2□ 每周 1-3 次　3□ 每月一次或更少

12. 请描述您给孩子吃或喝的食物的稠厚度：1□ 水样/非常薄　　2□ 稀粥样　　3□ 稠厚

13.请问您何时开始给孩子添加稀粥样食物：1□ 未添加　2□ 0～3 月　3□ 4～5 月　4□ 6～7 月　5□ 8～9 月

14. 请问您何时开始给孩子添加稠厚样食物：1□ 未添加　2□ 0～3 月　3□ 4～5 月　4□ 6～7 月　5□ 8～9 月

您过去的一周是否给孩子吃过以下的食物，多久吃一次？（总共吃过　　种食物组）

15. □ 米粉、米饭、面条	1□ 几乎每天	2□ 每周 1-3 次	3□ 每月一次或更少	4□ 没吃过
16. □ 玉米、粟米、小米	1□ 几乎每天	2□ 每周 1-3 次	3□ 每月一次或更少	4□ 没吃过
17. □ 各种蔬菜	1□ 几乎每天	2□ 每周 1-3 次	3□ 每月一次或更少	4□ 没吃过
18. □ 各种水果	1□ 几乎每天	2□ 每周 1-3 次	3□ 每月一次或更少	4□ 没吃过
19. □ 猪肉或鸡鸭肉	1□ 几乎每天	2□ 每周 1-3 次	3□ 每月一次或更少	4□ 没吃过
20. □ 肝脏或其他动物内脏	1□ 几乎每天	2□ 每周 1-3 次	3□ 每月一次或更少	4□ 没吃过
21. □ 鱼虾蟹	1□ 几乎每天	2□ 每周 1-3 次	3□ 每月一次或更少	4□ 没吃过
22. □ 牛奶或其他动物奶	1□ 几乎每天	2□ 每周 1-3 次	3□ 每月一次或更少	4□ 没吃过
23. □ 鸡蛋	1□ 几乎每天	2□ 每周 1-3 次	3□ 每月一次或更少	4□ 没吃过
24. □ 奶酪	1□ 几乎每天	2□ 每周 1-3 次	3□ 每月一次或更少	4□ 没吃过
25. □ 婴儿奶粉	1□ 几乎每天	2□ 每周 1-3 次	3□ 每月一次或更少	4□ 没吃过
26. □ 花生/其他坚果类	1□ 几乎每天	2□ 每周 1-3 次	3□ 每月一次或更少	4□ 没吃过
27. □ 豆类或豆制品	1□ 几乎每天	2□ 每周 1-3 次	3□ 每月一次或更少	4□ 没吃过
28. 其他 ______________	1□ 几乎每天	2□ 每周 1-3 次	3□ 每月一次或更少	4□ 没吃过

29. 您从孩子多大时开始添加奶类食品（奶粉、牛奶、奶酪等）？
1□ 未添加　2□ 0~3 月　3□ 4~5 月　4□ 6~7 月　5□ 8~9 月

30. 您从孩子多大时开始添加谷物类食品？
1□ 未添加　2□ 0~3 月　3□ 4~5 月　4□ 6~7 月　5□ 8~9 月

31.您从孩子多大时开始添加蔬菜类食品？
1□ 未添加　2□ 0~3 月　3□ 4~5 月　4□ 6~7 月　5□ 8~9 月

32.您从孩子多大时开始添加水果类食品？
1□ 未添加　2□ 0~3 月　3□ 4~5 月　4□ 6~7 月　5□ 8~9 月

 上海市 5-7 月龄婴儿辅助食品添加调查 地区[________________________]

代码: |___|___|___|___|___|___|

电话： 地址 [________________________]

33.您从孩子多大时开始添加蛋类食品？

1☐ 未添加 2☐ 0~3 月 3☐ 4~5 月 4☐ 6~7 月 5☐ 8~9 月

34. 您从孩子多大时开始添加肉类食品？

1☐ 未添加 2☐ 0~3 月 3☐ 4~5 月 4☐ 6~7 月 5☐ 8~9 月

35. 您在孩子的食物中加盐吗? 1☐ 是 2☐ 没有 → 跳到个问题.37

36. 您给孩子吃的是加碘盐吗? 1☐ 是 2☐ 没有 3☐ 不清楚

37. 您的孩子经常服用维生素和微量元素的补充剂吗? 1☐ 是 2☐ 没有→ 跳到第三部分

38. 您孩子使用的是哪种维生素和微量元素的补充剂?是从哪儿得到的？在下列表格中列出其来源

种类	医院处方	自己购买	不清楚
1．铁			
2．锌			
3．钙			
4．维生素 A			
5．维生素 D			
6．多种维生素			

第三部分：体格测量

测量两次——不要取平均值

1. **体重 (kg)**: 如果两次测量相差 10 克以上，测量第三次。

a. 第一次测量：|___|___|.|___|___| kg

b. 第二次测量：|___|___|.|___|___| kg

c. 第三次测量：|___|___|.|___|___| kg

d. 记录孩子衣服的重量：|___|___|.|___|___| kg

2. **头围 (cm)**: 如果两次测量相差 0.2 厘米以上测量第三次

a. 第一次测量：|___|___|. |___| cm

b. 第二次测量：|___|___|. |___| cm

c. 第三次测量：|___|___|. |___| cm

3. **身长 (cm)**: 如果两次测量相差 0.4 厘米以上测量第三次

a. 第一次测量：|___|___|. |___| cm

b. 第二次测量：|___|___|. |___| cm

c. 第三次测量：|___|___|. |___| cm

第四部分：表格完整性

1a. 填表者 |___|___|___|

1b. 填表日期: |__|__|__|__|-|__|__|-|__|__|

2a. 数据录入者 |___|___|___|

2b. 数据录入日期: |__|__|__|__|-|__|__|-|__|__|
