# Supplementary material for: Association between feeding practices and weight status in young children
Source: BMC Pediatr. 2015 Aug 26;15:97. doi: 10.1186/s12887-015-0418-4 (PMC4550067; doi:10.1186/s12887-015-0418-4)
Supplement: Additional file 2: — The follow-up questionnaire. (PDF 107 kb) [file 12887_2015_418_MOESM2_ESM.pdf]

表一：喂养情况

A 部分 喂养调查

1、孩子的主要喂养者是：<br>1 □母亲　2 □父亲　3 □祖母　4 □祖父　5 □外祖母　6 □外祖父<br>7 □保姆　8 □其他：______
2、您现在仍给宝宝喂母乳吗？　1 □是→ 跳至第 2 题　2 □否 → 跳至第 5 题
3、每天喂母乳的次数________次/天
4、您准备给宝宝喝母乳至他/她多大？　1 □12~18 月　2 □19~24 月　3 □大于 24 月
5、您的宝宝是什么时候断乳的?<br>1 □从来没有喂过　2 □0~3 月　3 □4~6 月　4 □7~9 月<br>5 □10~12 月　6 □13~月
6、您给宝宝喝白开水还是添加果汁、蜂蜜的水？<br>1 □白开水　2 □果汁水　3 □蜂蜜水　4 □ 其他（请详述_____________）
7、您每天大约给孩子喂几次奶? |___|___|　约_________ml
8、您的孩子每天吃正餐________次；点心________次；共________次
9、您孩子正餐食用的食物为：1□水样　2□稀粥样　3□稠厚　4□固体
10、请问您何时开始给孩子添加稀粥样食物：　1□ 未添加　2□ 0～3 月　3□4～5 月<br>4□ 6～7 月　5□ 8～9 月　6□10~12 月　7□大于 12 月
11、请问您何时开始给孩子添加稠厚样食物：1□ 未添加　2□ 0～3 月　3□4～5 月<br>4□ 6～7 月　5□ 8～9 月　6□10~12 月　7□大于 12 月
12、请问您何时开始给孩子添加固体食物：1□ 未添加　2□ 4～5 月　3□ 6~7 月　4<br>□8～9 月　5□ 10~12 月　6□ 13~15 月　7□ 16~18 月
13、最近一次血常规中血红蛋白含量：|__|__|__|g/L

您的孩子过去一周是否给吃过以下的食物，多久吃一次？（总共吃过__________种食物组）

11□各种蔬菜	1□几乎每天	2□每周 1-3 次	3□每月一次或更少	4□ 没吃过
12□玉米、粟米、小米	1□几乎每天	2□每周 1-3 次	3□每月一次或更少	4□ 没吃过
13□米粉、米饭、面条	1□ 几乎每天	2□每周 1-3 次	3□ 每月一次或更少	4□ 没吃过
14□各种水果	1□ 几乎每天	2□每周 1-3 次	3□ 每月一次或更少	4□ 没吃过
15□猪肉或鸡鸭肉	1□ 几乎每天	2□每周 1-3 次	3□ 每月一次或更少	4□ 没吃过
16□肝脏或其他动物内脏	1□ 几乎每天	2□每周 1-3 次	3□ 每月一次或更少	4□ 没吃过
17□鱼虾蟹	1□ 几乎每天	2□每周 1-3 次	3□ 每月一次或更少	4□ 没吃过
18□牛奶或其他动物奶	1□ 几乎每天	2□每周 1-3 次	3□ 每月一次或更少	4□ 没吃过
19□蛋类	1□ 几乎每天	2□每周 1-3 次	3□ 每月一次或更少	4□ 没吃过

20☐奶酪	1☐ 几乎每天	2☐每周 1-3 次	3☐ 每月一次或更少	4☐ 没吃过
21☐婴儿奶粉	1☐ 几乎每天	2☐每周 1-3 次	3☐ 每月一次或更少	4☐ 没吃过
22☐花生/其他坚果	1☐ 几乎每天	2☐每周 1-3 次	3☐ 每月一次或更少	4☐ 没吃过
23☐豆类或豆制品	1☐ 几乎每天	2☐每周 1-3 次	3☐ 每月一次或更少	4☐ 没吃过
24☐其他__________	1☐ 几乎每天	2☐每周 1-3 次	3☐ 每月一次或更少	4☐ 没吃过

B 部分 维生素、矿物质等添加情况

1.您孩子的食物中加盐吗？　1 ☐是　2 ☐否 → 跳至第 **3 题**

2.是加碘盐吗？　1 ☐是　2 ☐否

3.您的孩子常规服用维生素、矿物质或其他添加剂吗？1 ☐是　2 ☐否 → 跳至 **C 部分**

4.如果您的孩子服用，那么服用的是哪种，哪里买的？请在下面标出（√）添加剂的种类和来源。

种类	医疗机构	自己购买	不知道来源	不适用
a. 多种维生素				
b. 铁				
c. 锌				
d. 钙				
e. 维生素A				
f. 维生素D				
g. 其他				

C 部分. 人体测量

所有的测量都重复两遍——不要平均。

1. **体重**（g）：如果两次测量的差值大于0.1kg，进行第三次测量。

a. 第一次测量：|__|__|.|__|__|kg　b. 第二次测量：|__|__|.|__|__|kg
c. 第三次测量：|__|__|.|__|__|kg

2.**头围**（cm）：如果两次测量的差值大于0.2cm，进行第三次测量。

a. 第一次测量：|__|__|.|__|cm　b. 第二次测量：|__|__|.|__|cm
c. 第三次测量：|__|__|.|__|cm

3.**身长**（cm）：如果两次测量的差值大于0.4cm，进行第三次测量。

a. 第一次测量：|__|__|.|__|cm　b. 第二次测量：|__|__|.|__|cm
c. 第三次测量：|__|__|.|__|cm

D 部分.完成

1a.完成表格者的姓名：|__|__|__|　1b.完成表格的日期：|__|__|-|__|__|-|__|__|

2a.复核表格者的姓名：|__|__|__|　2b.复核表格的日期：|__|__|-|__|__|-|__|__|

3a.输入数据者的姓名：|__|__|__|　3b.输入数据的日期：|__|__|-|__|__|-|__|__|
