# Supplementary material for: Association between feeding practices and weight status in young children
Source: BMC Pediatr. 2015 Aug 26;15:97. doi: 10.1186/s12887-015-0418-4 (PMC4550067; doi:10.1186/s12887-015-0418-4)
Supplement: Additional file 3: — The young child feeding questionnaire (YCFQ). (PDF 122 kb) [file 12887_2015_418_MOESM3_ESM.pdf]

表二：喂养习惯

		从不	偶尔	较少	经常	总是	选项
1	您给宝宝喂食时，他/她会自己离开餐桌并到处走动吗？	1	2	3	4	5	[ ]
2	您的宝宝在进食时安静吗？	1	2	3	4	5	[ ]
3	您会给您的宝宝吃膨化食品、糖、碳酸饮料、油炸食品吗？	1	2	3	4	5	[ ]
4	您的宝宝偏爱高热量食物吗？（如甜食、巧克力、炸薯片等）	1	2	3	4	5	[ ]
5	您的宝宝偏爱淀粉类食物吗？（如饼干、蛋糕、面包、土豆）	1	2	3	4	5	[ ]
6	您的宝宝偏爱某种口味的食物吗？（如甜、咸、酸、其他＿＿＿＿＿）	1	2	3	4	5	[ ]
7	您会面对面给宝宝喂饭吗？	1	2	3	4	5	[ ]
8	您会追着宝宝喂饭吗？（他/她到处走动）	1	2	3	4	5	[ ]
9	如果宝宝自己吃得不够多，你会改用喂食的方式以让他/她吃得更饱？	1	2	3	4	5	[ ]
10	在喂食时，您会经常对您的宝宝微笑吗？	1	2	3	4	5	[ ]
11	在喂食时，您和您的宝宝会进行目光交流吗？	1	2	3	4	5	[ ]
12	在给宝宝喂食时或喂食前，您会为宝宝描述或赞美所吃的食物吗？	1	2	3	4	5	[ ]
13	您给宝宝喂食是一场斗争吗？	1	2	3	4	5	[ ]
14	您在意您的宝宝在进食期间将自己的身体、衣服或周围环境弄脏吗？	1	2	3	4	5	[ ]
15	当您给宝宝喂食时，您会对她说话或唱歌吗？	1	2	3	4	5	[ ]
16	您是否对孩子的进食行为或进食技能予以表扬？	1	2	3	4	5	[ ]
17	在进食时，您会采用哄骗或转移宝宝的注意力（如看电视、玩玩具）来增加其进食量吗?	1	2	3	4	5	[ ]
18	您给宝宝进食的位置固定吗？（饭桌固定的座位或宝宝专用的小桌）	1	2	3	4	5	[ ]
19	您会担心您的宝宝吃得太多吗？	1	2	3	4	5	[ ]
20	如果您的宝宝吃得太多，您会觉得不安吗？	1	2	3	4	5	[ ]
21	当宝宝饿了，您能很快察觉到吗？	1	2	3	4	5	[ ]
22	当宝宝吃饱了，您能很快察觉到吗？	1	2	3	4	5	[ ]
23	您会担心宝宝超重或肥胖吗？	1	2	3	4	5	[ ]
24	您会给宝宝限制食量以防止他/她超重吗？	1	2	3	4	5	[ ]
25	您会担心您的宝宝吃得不够吗？	1	2	3	4	5	[ ]
26	无论您给宝宝提供多少食物，他/她都能吃完吗？	1	2	3	4	5	[ ]
27	如果宝宝拒绝进餐，您会强迫他/她继续吃完吗（坚持要宝宝吃完一定量的食物）？	1	2	3	4	5	[ ]
28	如果您的宝宝有一顿不肯吃或者吃得不够多，您会在半小时内另外准备其他替代食物或点心给他/她吃吗？	1	2	3	4	5	[ ]
29	您的宝宝正餐和吃零食时间相对固定吗？	1	2	3	4	5	[ ]
30	当您的宝宝变得烦躁或哭吵时，即使没到吃饭时间，您会马上给他/她吃东西吗？	1	2	3	4	5	[ ]
31	如果您的宝宝吃得不够，您会觉得不安吗？	1	2	3	4	5	[ ]
32	为了让他/她睡得更久，您会给宝宝在睡前吃得饱一些吗？	1	2	3	4	5	[ ]
33	如果现在您的宝宝半夜醒来，您还会给他/她喝奶吗？	1	2	3	4	5	[ ]
34	当看到同龄的孩子比您宝宝胖时，您会觉得没喂好他/她吗？	1	2	3	4	5	[ ]

35	您认为如果不鼓励宝宝吃，他/她就会吃得不够多吗？	1	2	3	4	5	[ ]
36	您认为给您宝宝进食是让他/她停止烦躁或哭吵的最佳方法吗？	1	2	3	4	5	[ ]
37	您认为宝宝必须吃完每餐提供给他的食物吗？	1	2	3	4	5	[ ]
38	您会担心宝宝体重过轻吗？	1	2	3	4	5	[ ]
